# Response by Albanese et al to “Peristaltic Pump–Assisted Fat Harvesting and Closed-Circuit Grafting: Technique and Immediate Cell Membrane Integrity in a Case Series”

**DOI:** 10.1093/asjof/ojag198

**Published:** 2026-10-06

**Authors:** Damiano Tambasco, Roberta Albanese, Federica Tomaselli

We read with interest the article by Nichols and Albershardt describing peristaltic pump–assisted fat harvesting and closed-circuit grafting.^1^ Their discussion of aspiration pressure, tubing diameter, and flow provides a useful framework for evaluating the technique. We suggest that future evaluations also characterize the harvesting cannula, including its internal lumen and aperture geometry.

The authors report pressure measurements on the suction side of the circuit and describe the tubing used but do not provide comparable measurements at the harvesting cannula.^1^ Cannula dimensions and port configuration could influence resistance to flow and the way adipose tissue enters the aspiration pathway. A pressure measurement elsewhere in the circuit may therefore be insufficient to characterize conditions at the cannula–tissue interface.

At a given flow rate, a smaller effective opening would be expected to increase average local flow velocity. In practice, flow rate may also change as resistance increases. The resulting tissue deformation and cellular effects cannot be established from cannula dimensions or circuit pressure alone.

In a controlled experimental model using the same aspiration pressure, Kirkham et al reported greater graft retention and more favorable histologic findings after harvesting with a 5 mm rather than a 3 mm cannula.^2^ Subsequent correspondence discussed the interpretation of these findings.^3^ Lee et al found that higher shear during fat injection adversely affected graft outcomes, and subsequent correspondence further considered the interaction between pressure and shear.^4,5^ These publications support examining mechanical forces throughout fat transfer, but they do not establish how cannula design affects tissue integrity in the peristaltic harvesting system described by Nichols and Albershardt.

Future comparisons of peristaltic and conventional harvesting could report cannula lumen diameter, port area and geometry, flow rate, and the location of pressure measurements. Simultaneous assessment of these variables and prespecified measures of tissue integrity would help test whether cannula design contributes to differences between systems. Nichols and Albershardt have advanced a quantitative discussion of fat harvesting; including the cannula in that evaluation could make the proposed mechanical model more complete.
